# Measurement of sarcopenia in lung cancer inpatients and its association with frailty, nutritional risk, and malnutrition

**DOI:** 10.3389/fnut.2023.1143213

**Published:** 2023-04-17

**Authors:** Fang Wang, Hong-nan Zhen, Han-ping Wang, Kang Yu

**Affiliations:** ^1^Department of Clinical Nutrition, Peking Union Medical College Hospital, Chinese Academy of Medical Sciences and Peking Union Medical College, Beijing, China; ^2^Department of Radiotherapy, Peking Union Medical College Hospital, Chinese Academy of Medical Sciences and Peking Union Medical College, Beijing, China; ^3^Department of Respiratory and Critical Care Medicine, Peking Union Medical College Hospital, Chinese Academy of Medical Sciences and Peking Union Medical College, Beijing, China

**Keywords:** sarcopenia, frailty, nutritional risk, malnutrition, lung cancer

## Abstract

**Background:**

Sarcopenia, frailty, and malnutrition are associated with undesirable clinical outcomes in cancer patients. Sarcopenia-related measurements may be promising fast biomarkers for frailty. Our objectives were to assess the prevalence of nutritional risk, malnutrition, frailty, and sarcopenia in lung cancer inpatients, and describe the relationship of them.

**Methods:**

Stage III and IV lung cancer inpatients were recruited before chemotherapy. The skeletal muscle index (SMI) was assessed by multi-frequency bioelectric impedance analysis (m-BIA). Sarcopenia, frailty, nutritional risk, and malnutrition were diagnosed according to the Asian Working Group for Sarcopenia 2019 (AWGS 2019), Fried Frailty Phenotype (FFP), nutritional risk screening-2002 (NRS-2002), and Global Leadership Initiative on Malnutrition criteria (GLIM), and correlation analysis was performed between them with Pearson’s *r* correlation coefficients. A univariate and multivariate logistic regression analysis was conducted for all patients, gender and age-stratified subgroups to obtain odds ratios (ORs) and 95% confidence intervals (95%CIs).

**Results:**

The cohort included 97 men (77%) and 29 women (23%), with mean age of 64.8 ± 8.7 years. Among the 126 patients, 32 (25.4%) and 41 (32.5%) had sarcopenia and frailty, and the prevalence of nutritional risk and malnutrition was 31.0% (*n* = 39) and 25.4% (*n* = 32). Adjusted for age and gender, SMI was correlated with FFP (*r* = −0.204, *p* = 0.027), and did not remain significantly when stratified by gender. Stratification according to age revealed in ≥65-years-old population, SMI and FFP were significantly correlated (*r* = −0.297, *p* = 0.016), which is not seen in <65-years-old group (*r* = 0.048, *p* = 0.748). The multivariate regression analysis showed FFP, BMI, and ECOG were the independent variables associated with sarcopenia (OR 1.536, 95%CI 1.062–2.452, *p* = 0.042; OR 0.625, 95%CI 0.479–0.815, *p* = 0.001; OR 7.286, 95%CI 1.779–29.838, *p* = 0.004).

**Conclusion:**

Comprehensively assessed sarcopenia is independently associated with frailty based on FFP questionnaire, BMI, and ECOG. Therefore, sarcopenia assessment including m-BIA based SMI, and muscle strength and function could be used to indicate frailty to help select the targeting patients for care. Moreover, in addition to muscle mass, muscle quality should not be ignored in clinical practice.

## Introduction

Lung cancer is one of the most common malignancies with the highest number of new cases and the highest mortality rate in China (1). Chemotherapy, radiotherapy, and immunotherapy are the main treatment modalities that improve patient survival and quality of life (2). Among all cancer types, lung cancer has the third highest malnutrition rate at 38% (3–5). As the realization of the importance of muscle, reduced skeletal muscle mass could be a marker for malnutrition, and is proven to be associated with increased incidence of antineoplastic therapy-induced toxicity, decreased survival, and poor clinical outcomes in cancer patients (6–10). It has been found the cumulative recurrence rate at 5 years after surgery was significantly higher in NSCLC patients with sarcopenia than in patients without sarcopenia (49.9 and 22.4%, respectively), suggesting sarcopenia (OR 2.52, *p* = 0.001) an independent risk factor for postoperative recurrence (11). Under the effect of the disease, unreasonable diet, and reduced activity, there may be fat gain, masking the decline in muscle and weight, so monitoring weight alone cannot adequately reflect the nutritional risk. Instead, early identification of sarcopenia makes it possible to detect nutritional risk earlier and conduct an intervention to reduce chemo-and radio-therapy toxicity and improve clinical outcomes.

Many prior studies retrospectively measured skeletal muscle area (SMA) at the L3 level of abdominal CT scan as a mean of assessing sarcopenia (6, 9, 10), however, the diagnosis of sarcopenia in either European Working Group on Sarcopenia in Older People (EWGSOP) (12) or Asian Working Group for Sarcopenia 2019 (AWGS 2019) (13) includes a comprehensive assessment of muscle mass, strength, and function, so imaging alone does not constitute a completed diagnostic element. In addition, there is a lack of universally accepted thresholds for SMI due to sample size and different ethnicities, so the SMI cut-off values published in the previous literature are often based on the lowest quartile of the target population.

Nutritional risk screening is used to find patients who may be at nutritional risk and to perform subsequent nutritional care. Recently, GLIM criteria have been used to evaluate malnutrition in oncology patients. The prevalence of malnutrition diagnosed according to GLIM in lung cancer patients is as high as 47.5%, and there is a significant relationship between malnutrition with early cessation of anti-cancer therapy, mortality, and quality of life (14). Moreover, neutrophil-to-lymphocyte ratio (NLR) is widely used as a prognostic marker for inflammation, progression free survival (PFS), and overall survival (OS) in cancers (15–17).

Both the nutrition risk screening-2002 (NRS-2002) and the GLIM malnutrition assessment focus on recent weight loss, reduced food intake, low BMI, and disease burden, whereas GLIM also includes an assessment of muscle mass. Frailty refers to the patient’s vulnerability to the environment, and together with sarcopenia, it also involves a decrease in muscle strength and function. In addition, frailty focuses on the patients’ subjective perception of fatigue, whereas sarcopenia pays more attention to the objective assessment of muscle mass, however, both of which neither consider the recent decline in dietary intake nor the burden of disease aspects. Many previous studies have focused on nutrition-related assessments in lung cancer patients, but there is a lacking of comprehensive assessment of above indicators and their interrelationships. Moreover, there is a lack of an optimized and brief nutritional assessment process concerning nutritional risk, frailty, and muscle status for lung cancer inpatients. Therefore, this study is to investigate the prevalence of sarcopenia in pre-treatment lung cancer inpatients and analyzes its association with frailty and other related factors. Nevertheless, we also explore the relationship between sarcopenia, nutritional risk, malnutrition, and frailty.

## Materials and methods

### Subjects

This study was performed in Peking Union Medical College Hospital between December 2021 and March 2022. The inclusion criteria for this prospective study were as follows: (1) ≥18 years of age without gender limitation, (2) radiologically or pathologically diagnosed stage III-IV lung cancer within the past half year, (3) planned or initiated chemotherapy or chemoradiotherapy with/without immunotherapy, (4) could complete body composition analysis, handgrip strength, 6-M step speed, and questionnaires, (5) had not received nutritional support or professional guidance on dietary intake before admission, (6) intended to participate in this study voluntarily. The patients who had previously experienced chemotherapy, were with comorbid neuro-muscular related diseases (such as myasthenia gravis, paralysis, or Parkinson’s disease), suffered from severe medical diseases (such as stroke, liver and kidney failure, uncontrolled diabetes mellitus, hyperlipidemia, etc.), or were with the presence of drastic changes in body composition in the last 3 months (such as dehydration, persistent fever, edema, etc.), would be excluded.

### Ethics

The study was approved by the accredited Medical Research Ethics Committee in Peking Union Medical College Hospital (no. ZS-3321), and the study procedures were conducted following the Declaration of Helsinki. This study was registered on the Clinical Trial (NCT02873676). All patients provided written informed consent to participate in this study.

### Evaluation methods and data collection

Frailty was assessed by the Fried Frailty Phenotype (FFP), consisting of 5 phenotypes: unexplained weight loss, fatigue, decreased grip strength, decreased walking speed, and decreased physical activity, with a score of 0 considered healthy, 1–2 as pre-frailty, and ≥3 as frailty (18). Nutritional risk and malnutrition were assessed by the NRS-2002 and GLIM, respectively (Table 1).

**Table 1:** Domains and corresponding screening tools with applied cut-off values (13, 14, 17).

	Tests used	Outcome	Cut-off value
Muscle mass	m-BIA	SMI in cm^2^/m^2^	Male SMI <7.0 kg/m^2^
			Female SMI <5.7 kg/m^2^
Muscle strength	Handgrip	Kilogram	Male ≤28 kg, Female ≤18 kg
Muscle function	Walking speed	Meter/second	<1.0 m/s
Frailty	FFP	Score ranged 0–5	Healthy = 0
			Pre-frailty = 1–2
			Frailty ≥3
Nutritional risk	NRS-2002	Score ranged 0–7	No risk = NRS-2002 <3
Malnutrition	GLIM	Status	At least 1 phenotypic criterion and 1 etiologic criterion for diagnosis of malnutrition

m-BIA, multi-frequency bioelectric impedance analysis; FFP, fried frailty phenotype; GLIM, Global Leadership Initiative on Malnutrition Criteria; NRS-2002, Nutritional Risk Screening-2002.

Sarcopenia was diagnosed according to AWGS 2019 criteria. Skeletal muscle mass (SMM) was measured by multi-frequency bioelectric impedance analysis (m-BIA) at fasting state on the second morning after patient’s admission. To increase the accuracy of muscle mass assessment, we randomly selected 32 enrolled patients (25.4%) to assess the cross-sectional area (cm^2^) at the L3 level of abdominal CT scans within 2 weeks before admission, which have emerged as the golden standard (19). Segmentation of skeletal muscle, including the psoas, erector spinae, quadratus lumborum, transversus abdominis, external and internal obliques, and rectus abdominis muscles, was manually performed by Varian Eclipse, using a muscle-specific Hounsfield Unit (HU) range between −29 and +150. Two consecutive images were analyzed to generate the mean of SMA at L3. SMM was calculated according to the following formula (20) and normalized for patient’s height to calculate SMI (kg/m^2^). Then SMI obtained by the m-BIA and CT was compared. The handgrip strength and 6-M walking speed measurements were used to assess the patients’ muscle strength and function.

Total body muscle mass (kg) = 0.3 × skeletal muscle at L3 (cm^2^) + 6.06.

### Statistics

The baseline characteristics of patients were described. Continuous variables were described using means ± standard deviations (SD), and medians and quartiles for normally and non-normally distributed data, respectively. Ordinal or nominal variables were expressed as absolute values and percentages. Bivariate Pearson’s *r* correlation coefficients were used to evaluate the uniformity of SMM measurement by m-BIA and CT, and to analyze the correlation between SMI, sarcopenia, frailty, NRS-2002, and GLIM. Univariate logistic regression analyses were performed to assess whether frailty, nutritional risk, and malnutrition were related to muscle status, with sarcopenia as the dependent variable and baseline variables as independent variables. Possible multicollinearity was analyzed with variance inflation factor (VIF). Variables that were with statistical significance (*p* < 0.10) in univariate regression with VIF <3 were included in the multivariate logistic regression analysis in a backward manner. The strength of association between the variables and sarcopenia was expressed as odds ratios (OR) with corresponding 95% confidence intervals (CI). In the multivariate analysis, a *p* < 0.05 was considered statistically significant. SPSS version 26.0 (IBM SPSS Statistics) was used for statistical analysis.

## Results

### Demographic characteristics

The average age of the participants was 64.8 ± 8.7 years (range 34–86 years), and 77.0% were male. Pathological types included adenocarcinoma, squamous carcinoma, and small cell lung cancer, with the majority being stage IV (55.5%). Nutritional risk, malnutrition, frailty, sarcopenia (by AWGS 2019), handgrip strength, walking speed, and calf circumference were performed in all patients and shown in Table 2.

**Table 2:** Characteristics of demography and screening tools in participants with and without sarcopenia.

	Total (*n* = 126)	Non-sarcopenia (*n* = 94, 74.6%)	Sarcopenia (*n* = 32, 25.4%)	*p* value
Gender				0.642^*^
Female	29 (23.0%)	23 (24.5%)	6 (18.8%)	
Male	97 (77.0%)	71 (75.5%)	26 (81.3%)	
Age (years)	64.8 ± 8.7	63.8 ± 8.8	67.6 ± 7.6	**0.031**
<65	53 (42.1%)	44 (46.8%)	9 (28.1%)	
≥65	73 (57.9%)	50 (53.2%)	23 (71.9%)	
BMI (kg/m^2^)	24.0 ± 3.1	24.7 ± 2.8	21.7 ± 2.7	**<0.001**
<18.5	5 (4.0%)	1 (1.1%)	4 (12.5%)	
18.5–23.9	58 (46.0%)	36 (38.3%)	22 (68.8%)	
≥24.0	63 (50.0%)	57 (69.6%)	6 (18.8%)	**0.001**
aCCI	2.8 ± 1.4	2.6 ± 1.3	3.3 ± 1.4	**0.007**
0–1		18 (19.1%)	3 (9.4%)	
2–3		60 (63.8%)	15 (46.9%)	
≥4		16 (17.0%)	14 (43.8%)	
Smoking				0.293^*^
Never	39 (31.0%)	30 (31.9%)	9 (28.1%)	
Active/quit	87 (69.0%)	64 (68.0%)	23 (71.9%)	
SMI (kg/m^2^)	7.2 ± 0.9	7.5 ± 0.7	6.4 ± 0.8	**<0.001**
CC (cm)	34.6 ± 3.2	35.6 ± 2.4	31.7 ± 3.2	**<0.001**
VFA (cm^2^)	83.0 (60–108)	84.0 (66–112)	62.0 (50–106)	**0.024** ^**#**^
Handgrip (kg)	28.6 ± 8.1	30.0 ± 7.7	24.4 ± 7.9	**0.001**
Normal	82 (65.1%)	71 (75.5%)	11 (34.4%)	
Decreased	44 (34.9%)	23 (24.5%)	21 (65.6%)	**<0.001**
Walking speed (m/s)	1.2 ± 0.6	1.3 ± 0.3	1.0 ± 0.2	**0.004**
≥1.0	75 (59.5%)	65 (69.2%)	10 (31.3%)	<0.001
<1.0	51 (40.5%)	29 (30.9%)	22 (68.8%)	
Cancer histology				0.349^*^
Adenocarcinoma	47 (37.3%)	37 (39.4%)	10 (31.3%)	
Squamous-cell carcinoma	41 (32.5%)	30 (31.9%)	11 (34.4%)	
SCLC	38 (30.2%)	27 (28.7%)	11 (34.4%)	
Cancer stage				**0.016** ^*^
III	56 (44.4%)	48 (51.1%)	8 (25.0%)	
IV	70 (55.5%)	46 (48.9%)	24 (75.0%)	
NRS-2002	2.1 ± 1.2	1.9 ± 1.1	2.6 ± 1.4	**0.014**
<3	90 (71.4%)	69 (73.4%)	18 (56.3%)	
≥3	36 (28.6%)	25 (26.6%)	14 (43.8%)	
GLIM				**0.004**
Healthy	94 (74.6%)	73 (77.7%)	21 (65.6%)	
Malnutrition	32 (25.4%)	22 (23.4%)	10 (31.3%)	
Mild	26 (20.6%)	17 (18.1%)	9 (28.1%)	
Severe	6 (4.8%)	1 (1.1%)	5 (15.6%)	
NLR	4.3 ± 3.17	3.29 (2.47–4.55)	3.91 (2.64–5.15)	0.220^#^
FFP	4.6 ± 2.5	4.2 ± 2.2	5.8 ± 2.9	**<0.001**
Healthy	30 (23.8%)	30 (31.9%)	0	
Pre-frailty	55 (43.7%)	40 (42.6%)	15 (46.9%)	
Frailty	41 (32.5%)	24 (25.5%)	17 (53.1%)	
ECOG	1 (0–2)	0 (0–1)	1 (1–2)	**<0.001** ^*^
<2	86 (68.3%)	86 (91.5%)	20 (62.5%)	
≥2	20 (15.9%)	8 (8.5%)	12 (37.5%)	

aCCI, age-adjusted Charlson Comorbidity Index; BMI, body mass index; CC, calf circumference; ECOG, Eastern Cooperative Oncology Group; FFP, fried frailty phenotype; GLIM, Global Leadership Initiative on Malnutrition Criteria; NLR, neutrophil-lymphocyte ratio; NRS-2002, nutrition risk screening-2002; SCLC, small cell lung cancer; SMI, skeletal muscle index; VFA, visceral fat area. The non-normal distributed data are presented with median (interquartile range).

* *χ*^2^ test.

# Mann–Whitney *U*-test. Bold values denote statistical significance at the *p*<0.05 level.

According to the AWGS 2019 criteria, the prevalence of sarcopenia was 25.4% (*n* = 32), 20.7% (*n* = 6) in females and 26.8% (*n* = 26) in males. If the assessment was based on SMI alone, then 48 patients (38.1%) had reduced muscle mass (myopenia) with a mean SMI of 7.2 ± 0.9 kg/m^2^ in all, and 7.5 ± 0.7 kg/m^2^ and 6.4 ± 0.7 kg/m^2^ in males and females, respectively (*p* < 0.001). When assessed according to muscle strength or function decline, 43 patients (34.1%) had suspected probable sarcopenia, with a mean handgrip strength of 28.6 ± 8.1 kg and walking speed of 1.12 m/s. According to the FFP questionnaire, 32.5% (*n* = 41) and 43.8% (*n* = 55) of patients were in frailty and pre-frailty status.

Compared to non-sarcopenia patients, the sarcopenic group has elder age, lower BMI, more co-morbidities (aCCI ≥4), higher cancer stage (stage IV), higher prevalence of nutritional risk (NRS-2002 score ≥3), malnutrition, frailty, and ECOG (≥2) (*p* < 0.05), however, gender, pathological type, and NLR were not significantly different between two groups.

### The agreement of muscle mass measurements

Thirty-two patients were randomly selected for consistent evaluation of SMI measured by m-BIA and CT. SMI assessed by CT and calculated as 7.0 ± 1.4 kg/m^2^, which is comparable to m-BIA result (*r* = 0.791, *p* = 0.011), indicating the SMI measurement using the m-BIA method were reliable in this study.

### Correlation analysis of sarcopenia, nutritional risk, malnutrition, and frailty

After correcting for age, gender, and cancer stage, the correlations between sarcopenia, FFP score, NRS-2002 score, and GLIM classification are shown in Table 3. Sarcopenia and frailty were significantly correlated (*p* < 0.001) since both of which focus on muscle strength and function in their respective assessment criteria. Although muscle mass assessment is lacking in frailty, SMI was shown significantly correlated with frailty scores in this study (*r* = −0.204, *p* = 0.027). Although calf circumference, another measure of muscle mass, showed a negative correlation with frailty, was not statistically significant (*r* = −0.063, *p* = 0.602), because calf circumference may be influenced by body size and cannot accurately reflect SMM. Frailty is also strongly correlated with nutritional risk and malnutrition as they both include an evaluation of recent weight loss. Although the nutrition risk screening lacked an evaluation of muscle status, the correlation between SMI and NRS-2002 scores was significant (*r* = −0.230, *p* = 0.013), while handgrip strength and walking speed were not correlated with the NRS-2002 screen (*r* = −0.176, *p* = 0.057; r = −0.113, *p* = 0.212), which could explain the absent of significant correlation between nutritional risk and sarcopenia. Although GLIM did not focus on muscle strength and function, it was significantly correlated with sarcopenia (*r* = 0.436, *p* < 0.001), handgrip strength, and walking speed (*r* = −0.239, *p* = 0.008; *r* = −0.197, *p* = 0.030).

**Table 3:** Correlation among different evaluation criteria.

	NRS-2002		GLIM		Frailty		*r*	*p*	*r*	*p*	*r*	*p*
Sarcopenia	0.157	0.082	0.446	<0.001	0.335	<0.001
NRS-2002	–		0.525	<0.001	0.357	<0.001
GLIM	–		-		0.453	<0.001

GLIM, Global Leadership Initiative on Malnutrition Criteria.

**Figure 1:**
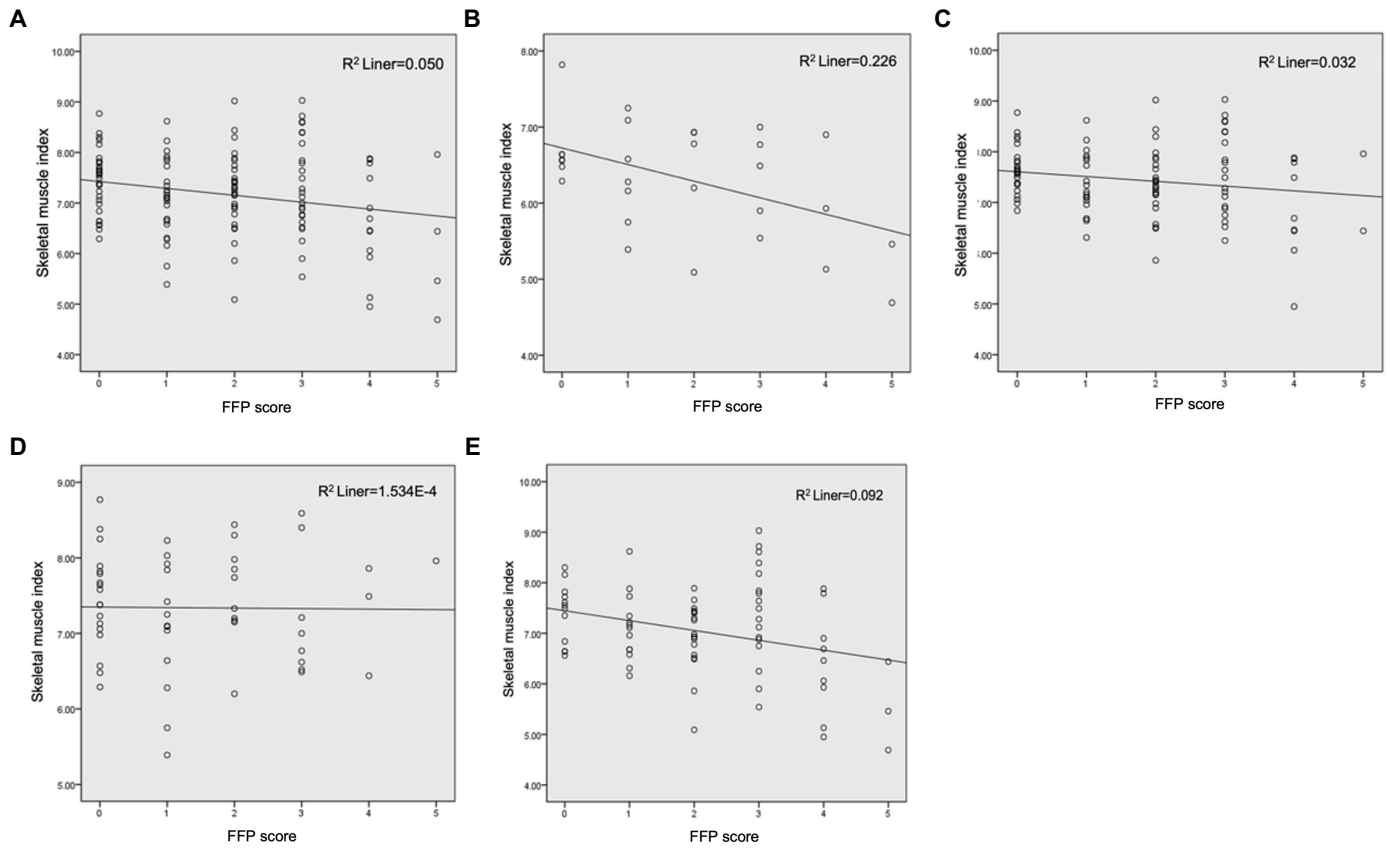
Correlation analysis of SMI and FFP. **(A)** Total subjects; **(B)** Female; **(C)** Male; **(D)** <65 years; **(E)** ≥65 years.

The scatterplots for SMI and FFP revealed a negative correlation for the whole population (Figure 1A). After correcting for age, gender, and cancer stage, the correlation analysis demonstrated both SMI and comprehensively assessed sarcopenia were significantly correlated with FFP scores in all populations (*r* = 0.335, *p* < 0.001; *r* = −0.215, *p* = 0.017). In addition, NRS-2002 and aCCI also showed a positive correlation with FFP (*r* = 0.357, *p* < 0.001; *r* = 0.348, *p* < 0.001). However. BMI, visceral fat, calf circumference, and NLR (*r* = −0.078, *p* = 0.406; *r* = 0.109, *p* = 0.245; *r* = −0.045, *p* = 0.629; *r* = 0.129, *p* = 0.157) were not associated with FFP.

Gender was stratified to clarify the effect of gender on the relationship between SMI and frailty scores. Although scatterplots still showed a negative correlation between SMI and FFP (Figures 1B,C), the correlation analysis, adjusted for age and cancer stage, suggested SMI was not significantly correlated with FFP in females and males (*r* = −0.213, *p* = 0.287; *r* = −0.098, *p* = 0.348), however FFP in men (*n* = 97) showed significant association with sarcopenia (*r* = −0.555, *p* < 0.001). Therefore, it suggested we cannot focus on muscle mass alone, but need to evaluate muscle strength and function to better predict frailty. In women, the intensity of the analysis may have been limited by the sample size.

Data were stratified for the age group to clarify the effect of age on the relationship between SMI and frailty, and scatterplots are illustrated in Figures 1D,E. In the <65-year-old population (*n* = 53), after correction for age, sex, and cancer stage, FFP was associated with sarcopenia (*r* = 0.325, *p* = 0.024), but not with SMI (*r* = 0.048, *p* = 0.748). In the ≥65-year-old population (*n* = 73), FFP was associated with both sarcopenia and SMI (*r* = 0.296, *p* = 0.017; *r* = −0.297, *p* = 0.016). This indicates that frailty is less related to baseline SMI in people <65 years old, and the close association of frailty and sarcopenia is more likely to be contributed by muscle strength and function, emphasizing the importance of the evaluation of both. However, in the ≥65 years old group, frailty was associated with both muscle mass and quality. Remarkably, in the <65 and ≥65 years old group, frailty was associated with aCCI (*r* = 0.380, *p* = 0.008; *r* = 0.351, *p* = 0.004), NRS-2002 (*r* = 0.341, *p* = 0.018; *r* = 0.398, *p* = 0.001), and malnutrition (*r* = 0.405, *p* = 0.004; *r* = 0.463, *p* < 0.001), indicating the comorbidities and nutritional status should be paid attention to.

### Univariate and multivariate logistic regression

Table 4 summarizes the univariate logistic regression analysis with sarcopenia as the dependent variable. More co-morbidities (aCCI ≥4), later cancer stage (stage IV), malnutrition, higher FFP score, and ECOG were risk factors for sarcopenia, whereas BMI ≥24.0 kg/m^2^ was a protective factor for sarcopenia (*p* < 0.001). Patients with sarcopenia tend to be male, older (≥65 years), smoking, with lower BMI (<18.5 kg/m^2^), with nutritional risk, and with higher NLR compared to patients without sarcopenia, but the association was not statistically significant.

**Table 4:** Univariate and multivariate logistic regression analysis.

Variables		Univariate logistic regression		Multivariate logistic regression		OR (95% CI)	*p* value	OR (95% CI)	*p* value
Gender	Female	1				Male	1.404 (0.514, 3.833)	0.508		
Age	<65	1				≥65	2.249 (0.942, 5.371)	0.068		
BMI (kg/m^2^)	18.5–23.9	1	**0.024**^**#**^	1	**0.017**^**#**^	<18.5	6.545 (0.687, 15.213)	0.102	6.095 (1.799, 13.649)	0.244	≥24.0	0.172 (0.064, 0.466)	**0.001**	0.088 (0.021, 0.372)	**0.042**
aCCI	0–1	1				2–3	1.500 (0.390, 5.768)	0.555	1.039 (0.175, 6.155)	0.966	≥4	2.291 (1.128, 4.654)	**0.022**	3.128 (0.449, 21.778)	0.249
Smoking	Never	1				Active/quit	1.198 (0.495, 2.900)	0.689		
Cancer histology	Adenocarcinoma	1			
	Squamous-cell carcinoma	1.553 (0.618, 3.901)	0.349			SCLC	1.234 (0.738, 2.063)	0.423		
Cancer stage	III	1				IV	3.000 (1.224, 7.353)	**0.016**		
NRS-2002	<3	1				≥3	2.147 (0.931, 4.947)	0.073		
GLIM	Healthy	1				Malnutrition	3.981 (1.531, 10.358)	**0.005**		
FFP		1.956 (1.402, 2.730)	**<0.001**	1.553 (1.030, 2.343)	**0.036**
ECOG	<2	1		1		≥2	6.450 (2.330, 17.858)	**<0.001**	7.286 (1.779, 29.838)	**0.006**
NLR		1.058 (0.938, 1.188)	0.366		

aCCI, age-adjusted Charlson Comorbidity Index; BMI, body mass index; CC, calf circumference; ECOG, Eastern Cooperative Oncology Group; FFP, fried frailty phenotype; GLIM, Global Leadership Initiative on Malnutrition Criteria; NLR, neutrophil-lymphocyte ratio; NRS-2002, nutrition risk screening-2002; SCLC, small cell lung cancer; SMI, skeletal muscle index; VFA, visceral fat area. The non-normal distributed data are presented with median (interquartile range). ^#^Overall *p* value of variable. Bold values denote statistical significance at the *p*<0.05 level.

The covariance test showed no covariance among the variables (VIF <2), so age, BMI, aCCI, NRS-2002 score, FFP, ECOG, cancer stage, and GLIM classification were put into the multivariate analysis (see Table 4). The results showed BMI (OR 0.551, 95%CI 0.338–0.897, *p* = 0.017), FFP score (OR 1.553, 95%CI 1.030–2.343, *p* = 0.036), and ECOG (OR 7.286, 95%CI 1.779–29.838, *p* = 0.004) were the influencing factors of sarcopenia.

We aimed to analyze both males and females, but the sample size of female was too small (*n* < 50). In males, univariate regression analysis with sarcopenia as a dependent variable was performed and found BMI (OR 0.678, 95%CI 0.546–0.842, *p* < 0.001), cancer stage (OR 3.627, 95%CI 1.302–10.103, *p* = 0.014), GLIM (OR 4.213, 95%CI 2.562–12.416, *p* = 0.001), and FFP (OR 1.876, 95%CI 1.284–2.740, *p* = 0.001) were significant variables. Multivariate regression analysis in a backward manner revealed FFP (OR 1.536, 95%CI 1.062–2.452, *p* = 0.042) and BMI (OR 0.625 95%CI 0.479–0.815, *p* = 0.001) as independent variables associated with sarcopenia in males (Table 5).

**Table 5:** Univariate and multivariate logistic regression analysis for male.

Variables		Univariate logistic regression		Multivariate logistic regression		OR (95% CI)	*p* value	OR (95% CI)	*p* value
BMI (kg/m^2^)		0.678 (0.546, 0.842)	**<0.001**	0.625 (0.479, 0.815)	**0.001**
Cancer stage	III	1				IV	3.627 (1.302, 10.103)	**0.014**		
GLIM	Healthy	1	0.001			Slight malnutrition	3.750 (1.300, 10.817)	**0.014**			Severe malnutrition	5.000 (4.603, 10.216)	**0.001**		
FFP		1.876 (1.284, 2.740)	**0.001**	1.536 (1.062, 2,452)	**0.042**

BMI, body mass index; FFP, fried frailty phenotype; GLIM, Global Leadership Initiative on Malnutrition Criteria. Bold values denote statistical significance at the *p*<0.05 level.

Univariate and multivariate logistic regression analysis was performed stratified by age. For both the <65 and ≥65 years old populations, BMI, GLIM, and FFP were significant variables for each subgroup (*p* < 0.05), while in a multivariate regression analysis in a backward manner, BMI and FFP were independent variables for sarcopenia (Tables 6, 7).

**Table 6:** Univariate and multivariate logistic regression analysis for subjects <65 years old.

Variables		Univariate logistic regression		Multivariate logistic regression		OR (95% CI)	*p* value	OR (95% CI)	*p* value
BMI (kg/m^2^)		0.521 (0.332, 0.818)	**0.005**	0.428 (0.228, 0.802)	**0.008**
NRS-2002	<3	1				≥3	4.229 (0.904, 19.786)	0.067		
GLIM	Healthy	1	0.137			Slight malnutrition	5.700 (1.030, 34.950)	**0.046**			Severe malnutrition		**0.032**		
FFP		2.172 (1.198, 3.941)	**0.011**	2.919 (1.299, 6.558)	**0.009**

BMI, body mass index; FFP, fried frailty phenotype; GLIM, Global Leadership Initiative on Malnutrition Criteria; NRS-2002, nutrition risk screening-2002. Bold values denote statistical significance at the *p*<0.05 level.

**Table 7:** Univariate and multivariate logistic regression analysis for subjects ≥65.

Variables		Univariate logistic regression		Multivariate logistic regression		OR (95% CI)	*p* value	OR (95% CI)	*p* value
BMI (kg/m^2^)		0.708 (0.571, 0.877)	**0.002**	0.719 (0.574, 0.900)	**0.004**
Cancer stage	III	1				IV	2.614 (0.885, 7.729)	0.082		
GLIM	Healthy	1	0.003			Slight malnutrition	3.094 (0.938, 10.201)	**0.046**			Severe malnutrition	44.000 (4.621, 78.927)	**0.001**		
FFP		1.769 (1.173, 2.668)	**0.007**	1.701 (1.095, 2.643)	**0.018**

BMI, body mass index; FFP, fried frailty phenotype; GLIM, Global Leadership Initiative on Malnutrition Criteria; NRS-2002, nutrition risk screening-2002. Bold values denote statistical significance at the *p*<0.05 level.

## Discussion

This manuscript described the prevalence and investigated the relationship of frailty, sarcopenia, nutritional risk, and malnutrition in 126 hospitalized patients with primary lung cancer. This is the first study that proposes that comprehensively assessed sarcopenia is independently associated with frailty based on FFP in lung cancer inpatients. The results suggest that comprehensive sarcopenia assessment, including muscle mass and quality, is more correlated to frailty, and comprehensive evaluation for sarcopenia may represent frailty status.

Studies over the past have shown that sarcopenia is prevalent in lung cancer patients and is associated with chemotherapy toxicity, tolerance, and short survival (21–24), emphasizing the importance of identifying the sarcopenic group. According to the AWGS 2019 criteria, the incidence of sarcopenia in our study was 25.4%, which was lower than 43–52% in some previous studies (25–27). Firstly, the patients of our studies were newly diagnosed and initially treated patients, so the shorter duration of illness and the lack of exposure to chemotherapy might be the reason. Secondly, most of the previous studies only reported CT-defined sarcopenia, instead of comprehensive sarcopenia. In this study, the incidence of decreased muscle mass based on SMI was 38.1%, which is also called muscle disorder or myopenia, however, some of this population may have an inherent insufficient of muscle mass, but not represent a strength or functional reduction.

Since patients need regular chest and abdomen CT scan to evaluate the treatment effect, most previous studies on sarcopenia in lung cancer retrospectively reported the SMA and attenuation at the L3 level of CT to describe muscle mass and quality. However, there is a lack of consensus on the standardized thresholds of CT-based SMI used for diagnosing sarcopenia. Nevertheless, in clinical practice, finding recent CT images and then asking imaging physicians to measure SMA and SMD using specialized software is more cumbersome. M-BIA is widely used in body composition assessment in clinics, and there is a strong correlation between its measurements and those assessed by CT (28, 29). Moreover, m-BIA detection is non-invasive, radiation-free, and quick. Therefore, the AWGS 2019 diagnostic criteria includes m-BIA as a method to assess SMM. M-BIA does not measure intermuscular fat or provide the insight into muscle quality, and is susceptible to the patient’s hydration status, but the patients in this study were in a fasting, moderately hydrated condition to reduce error. In addition, the actual measurement of handgrip strength and walking speed compensates for the assessment of muscle quality, which has a greater ability than muscle mass to predict poor outcome in sarcopenic patients (12), and can be integrated into the clinical pathway for inpatients, allowing better selection of patients to receive intensive therapy. For this reason, we evaluated sarcopenia strictly according to the AWGS 2019 criteria and demonstrated sarcopenia was closely related to FFP scores, and that FFP, BMI, and ECOG were influencing factors in sarcopenia.

Sarcopenia and frailty are partially overlapping but fundamentally different conditions. Frailty is defined as a state of the significant impact caused by stress; therefore its assessment focuses on psychology, cognitive function, family support, and the subjective feelings of the patients (30). Sarcopenia is a state of a progressive and generalized loss of skeletal muscle mass, strength, and function, and therefore focuses more on physical aspects (31). The FFP score (18) conceptualizes and quantifies weight loss and impaired mobility as potential factors of frailty (32), and sarcopenia uses handgrip strength and walking speed as measures, which may explain the accordance of the two conditions (33, 34). Our findings suggested a strong correlation between sarcopenia and frailty, but this result may be influenced by the evaluation criteria chosen. As demonstrated in a previous study of HNC patients (35), low SMI was associated with G8 scores but not with GFI, because GFI focuses more on the social and cognitive aspects of frailty. Although muscle mass is not addressed in the diagnostic criteria for FFP, we found a significant correlation between SMI and FFP. As known, muscle mass reduction is associated with a decrease in somatic activity function (34). As a result, our results confirm that frailty and sarcopenia are two intersecting and overlapping states in patients with lung cancer (36).

In performing subgroup analyses by gender and age, we found FFP and SMI were closely correlated only in the ≥65-year-old group, while in the <65-year-old and the male subgroup, FFP was only correlated with sarcopenia, but not SMI. Moreover, it has been shown that muscle quality, such as SMD on CT imaging, is more relevant to frailty in older patients than skeletal muscle quantification (33). As a result, muscle strength and function assessment cannot be ignored. The <65-year-old and male groups are with relatively good muscle mass at baseline, and their frailty is more likely to be affected by the disease. In the population aged ≥65 years, FFP was more frequently associated with SMI, sarcopenia, and malnutrition, indicating the importance of maintaining muscle mass and nutrition status in this population.

The prevalence of low BMI was only 4.0%, but the prevalence of inadequate muscle mass was as high as 38.1%, supporting earlier findings that muscle mass is not necessarily related to BMI (20, 37), because cancer patients may be accompanied by an increase of adipose tissue, so weight cannot be used alone to assess nutritional status. However, BMI is still an important influencing factor on sarcopenia since the regression analysis in this study found a larger BMI was a protective factor for sarcopenia, therefore it is important to positively improve the low body weight in oncology patients. Although GLIM was significantly correlated with both sarcopenia and frailty, it was not an influencing factor for sarcopenia in the regression analysis, probably because both NRS-2002 scores and GLIM focused less on muscle quality. This study showed that there was a significant correlation between sarcopenia and frailty, nutritional risk, and malnutrition in lung cancer patients, but only frailty, low BMI, and ECOG were risk factors for sarcopenia in the multivariable regression analysis, and such results did not differ between different gender age groups, indicating the results were not gender or age dependent.

Sarcopenia, malnutrition, and frailty can occur concurrently in cancer patients since they all include the assessment of nutrition status and muscle loss and are related to poor clinical outcomes. Cancer cachexia, proposed by an expert panel in 2012, was defined as a multifactorial syndrome manifested by an ongoing muscle loss (with or without adiposity loss), could not be fully reversed by conventional nutritional support, and contributes to functional impairment (38). The diagnostic criteria of cancer cachexia include malnutrition, loss of muscle mass, and abnormal biochemical markers related to inflammation and metabolic alternations. Since loss of muscle is the common characteristics shared by sarcopenia and cachexia, sometimes they are overlapping. However, cachexia also underlies involuntary weight loss, reduced food intake, and systematic inflammation, while sarcopenia emphasizes the objective manifestation and comprehensively qualitative and quantitative muscle assessment, instead of recent nutritional status alternations. Although sarcopenia was initially regarded as age-related, it has been recently found to be secondary to disease and related to adverse clinical outcomes and physical dysfunction. Moreover, in our study, age was not an influencing factor for cancer-related sarcopenia in lung cancer. Ideally, further studies could follow up the patients to collect changes in muscle mass, treatment-related toxicity, and survival to investigate the clinical outcome most correlated screening scales and influencing factors.

With the understanding of the effects of nutritional risk and muscle on clinical outcomes in lung cancer patients, nutritional intervention should be conducted. In patients with advanced cancer, maintaining skeletal muscle mass and physical function is often challenging and complicated due the anorexia and weakness. Usually, nutrition intervention, including sufficient calory intake, increased protein intake beyond 1.2 g/kg/day, and physical activities are recommended (39, 40), and some antioxidants, amino acid supplementation, and vitamin D may also help in muscle maintenance. However more studies are required to determine the optimal multimodal interventions and their impact on clinical outcomes in cancer patients.

Our study has several strengths. First, this study was of patients with pre-treatment lung cancer, who were not affected by prior treatment. Second, the prospective study collected comprehensive information on patients, including diet and weight changes, and comorbidities, which were more accurate than in the retrospective study. Third, considering the possible error of BIA on body composition assessment, 25% of the patients were selected randomly to compare the SMI results assessed by BIA and CT. In addition, according to the AWGS 2019 diagnostic criteria, BIA has a clear cut-off value for SMI and is homozygous for Asians. Fourth, we used rigorous criteria for sarcopenia diagnosis and comprehensively assessed muscle mass and quality.

The present study also has some limitations. First, as a cross-sectional study, it is unclear which screening modality or which of this screening questionnaire correlates best with clinical outcomes. Second, although patients were required to conduct BIA in a fasting, well-hydrated state, BIA is still vulnerable to individual differences and would be less inaccurate with BMI beyond the range of 16–34 kg/m^2^. Last, although the study included more than 100 patients, the sample size and unicentric characteristic still limited the generalization of the results, so our findings should also be tested in larger sample size and multicenter cohort studies.

## Conclusion

This study found a significant relationship between sarcopenia, frailty, nutritional risk, and malnutrition in 126 lung cancer hospitalized patients. Since sarcopenia has the potential to be a cost-effective, non-invasive biomarker for patients with frailty and malnutrition, screening for sarcopenia is useful to screen target patients for further nutrition support. Moreover, comprehensively assessment of muscle mass and quality is more correlated with frailty and should be conducted in clinical practice.

## Data availability statement

The raw data supporting the conclusions of this article will be made available by the authors, without undue reservation.

## Ethics statement

The studies involving human participants were reviewed and approved by Medical Research Ethics Committee in Peking Union Medical College Hospital. The patients/participants provided their written informed consent to participate in this study.

## Author contributions

FW, H-pW, and KY designed this study and revised the paper. FW and H-nZ conducted the research, analyzed the data, and wrote the manuscript. All authors contributed to the article and approved the submitted version.

## Funding

This study was funded by the National High Level Hospital Clinical Research Funding (no. 2022-PUMCH-B-055). The funding sources had no role in study design or conduction, data collection or analysis, or the writing of the report.

## Conflict of interest

The authors declare that the research was conducted in the absence of any commercial or financial relationships that could be construed as a potential conflict of interest.

## Publisher’s note

All claims expressed in this article are solely those of the authors and do not necessarily represent those of their affiliated organizations, or those of the publisher, the editors and the reviewers. Any product that may be evaluated in this article, or claim that may be made by its manufacturer, is not guaranteed or endorsed by the publisher.
